# Plasma *ESR1* mutations and outcome to first-line paclitaxel and bevacizumab in patients with advanced ER-positive/HER2-negative breast cancer

**DOI:** 10.1007/s10549-023-06965-5

**Published:** 2023-05-25

**Authors:** M. K. Bos, S. W. Lam, G. Motta, J. C. A. Helmijr, C. M. Beaufort, E. de Jonge, J. W. M. Martens, E. Boven, M. P. H. M. Jansen, A. Jager, S. Sleijfer

**Affiliations:** 1 Department of Medical Oncology, Erasmus MC Cancer Institute, Erasmus University Medical Center, Dr. Molewaterplein 40, 3015 GD Rotterdam, The Netherlands; 2 Department of Medical Oncology, Amsterdam UMC, Vrije Universiteit Amsterdam/Cancer Center Amsterdam, De Boelelaan 1117, 1081 HV Amsterdam, The Netherlands; 3 Department of Clinical Chemistry, Erasmus University Medical Center, Rotterdam, The Netherlands; 4 IOM (Mediterranean Institute of Oncology) Research, Viagrande, Catania, Italy; 5 Department of Clinical and Experimental Medicine, A.O.U. Policlinico-Vittorio Emanuele, Center of Experimental Oncology and Hematology, University of Catania, Catania, Italy; 6 Present Address: Department of Radiology, The Netherlands, Cancer Institute/Antoni Van Leeuwenhoek Hospital, Plesmanlaan 121, 1066 CX Amsterdam, The Netherlands

**Keywords:** HR +/HER2- advanced breast cancer, *ESR1* mutation, Paclitaxel, Bevacizumab, ctDNA

## Abstract

**Background:**

*ESR1* mutations have been identified as mechanism for endocrine resistance and are also associated with a decreased overall survival. We assessed *ESR1* mutations in circulating tumor DNA (ctDNA) for impact on outcome to taxane-based chemotherapy in advanced breast cancer patients.

**Methods:**

*ESR1* mutations were determined in archived plasma samples from patients treated with paclitaxel and bevacizumab (AT arm, *N* = 91) in the randomized phase II ATX study. Samples collected at baseline (*n* = 51) and at cycle 2 (*n* = 13, C2) were analyzed using a breast cancer next-generation sequencing panel. This study was powered to detect a benefit in progression-free survival (PFS) at six months for patients treated with paclitaxel/bevacizumab compared to historical trials with fulvestrant. PFS, overall survival (OS), and ctDNA dynamics were exploratory analyses.

**Results:**

PFS at six months was 86% (18/21) in patients with an *ESR1* mutation detected and 85% (23/27) in wildtype *ESR1* patients. In our exploratory analysis, median progression-free survival (PFS) was 8.2 months [95% CI, 7.6–8.8] for *ESR1* mutant patients versus 8.7 months [95% confidence interval (CI), 8.3–9.2] for *ESR1* wildtype patients [*p* = 0.47]. The median overall survival (OS) was 20.7 months [95% CI, 6.6–33.7] for *ESR1* mutant patients versus 28.1 months [95% confidence interval (CI), 19.3–36.9] for *ESR1* wildtype patients [*p* = 0.27]. Patients with ≥ two *ESR1* mutations had a significantly worse OS, but not PFS, compared to those who did not [p = 0.003]. Change in ctDNA level at C2 was not different between *ESR1* and other mutations.

**Conclusions:**

Presence of *ESR1* mutations in baseline ctDNA might not be associated with inferior PFS and OS in advanced breast cancer patients treated with paclitaxel/bevacizumab.

**Supplementary Information:**

The online version contains supplementary material available at 10.1007/s10549-023-06965-5.

## Introduction

In metastatic estrogen receptor-positive (ER) breast cancer (MBC), *ESR1* mutations have been identified as an important mechanism of resistance to estrogen deprivation therapy by aromatase inhibitors (AIs) and can be detected in approximately 30–50% of MBC patients after prior treatment with AIs in the metastatic setting [16, 20]. Circulating tumor DNA (ctDNA) is now often used as a non-invasive tool for the identification of *ESR1* mutations. Additionally, ctDNA is thought to represent the full metastatic landscape which is known to be heterogenic. ctDNA analysis might, therefore, overcome sampling bias introduced by biopsy of one single lesion.

How *ESR1* mutations affect sensitivity to subsequent treatment strategies is currently being investigated. Several studies have demonstrated that efficacy of the selective ER down-regulator (SERD) fulvestrant, which is often administered after occurrence of resistance to AIs, is not impaired in patients with *ESR1* mutations [6, 19, 21]. More recently, endocrine treatment is being combined with CDK4/6 inhibitors (CDK4/6-i) in the first or second line. Analyses of the PEARL and PALOMA-3 studies have suggested that also CDK4/6-i are effective in patients with *ESR1* mutations when combined with fulvestrant [6, 11]. Whether the efficacy of CDK4/6-i when combined with an AI is retained in patients with *ESR1* mutations is currently under debate [4, 11]. Also, novel SERDs, which have more favorable pharmacokinetics and ER down-regulating potential than fulvestrant, are being posed as treatment option for patients with *ESR1* mutant breast cancer. Elacestrant was recently approved by the FDA for patients an *ESR1* mutation who have previously received CDK4/6-I, based on a median PFS improvement of 1.9 months (standard of care endocrine monotherapy) to 3.8 months [3]. In an alternative approach, the PADA-1 trial investigated whether early switching of endocrine therapy based on the occurrence of an *ESR1* mutation in plasma, instead of radiological progression, improved outcomes. This study demonstrated that such an approach improves PFS from 5.7 months in patients remaining on AI treatment to 11.9 in patients switching from AI to fulvestrant [2]. However, it is unknown whether this PFS benefit translated into improved OS.

Next to the predictive value of *ESR1* mutations for outcome to treatment with AIs, *ESR1* mutations have also been associated with worse overall survival [5, 21]. This latter finding suggests that patients with *ESR1* mutations have a more aggressive disease biology. The optimal treatment for patients with *ESR1* mutations has, however, not been properly determined. For example, it is yet unknown how patients with *ESR1* mutations respond to chemotherapy. Therefore, we investigated the outcome to taxane-based chemotherapy in patients with an *ESR1* mutation. We analyzed baseline and follow-up plasma samples from the ATX study in which patients were treated with taxane-based chemotherapy after they progressed on endocrine treatment [8].

## Methods

### Patient population

The ATX study (BOOG-2006-06) was a multicenter, open-label, randomized phase II trial initiated in 2006. Women with confirmed HER2-negative locally recurrent or MBC were eligible. Patients were randomized between paclitaxel (day 1, 8, and 15) plus bevacizumab (days 1 and 15) (AT) at 4-week intervals for six cycles or paclitaxel (day 1 and 8) plus bevacizumab (day 1) plus capecitabine (ATX) at 3-week intervals for eight cycles. For this plasma analysis, we included samples from patients in the AT arm who received prior AI treatment in the adjuvant and/or metastatic setting. We only analyzed samples from the AT arm as the ATX trial showed a difference in progression-free survival (PFS) between the two treatment arms with a median PFS of 11.2 months in the ATX arm and 8.4 months in the AT arm [8]. Moreover, we selected only samples from patients who received an aromatase-inhibitor prior to inclusion. The study was approved by ethical or institutional review boards of the participating hospitals as described previously [8], conducted in agreement with the Declaration of Helsinki and registered with the European Union Drug Regulating Authorities Clinical Trial, number 2006-006058-83, and the Netherlands Trial Register, number NTR1348.

### ESR1 mutation analysis

In the trial, plasma was collected from consenting patients at baseline and on day 1 of cycle 2. DNA was isolated from the total amount of plasma that was available, being a median of 1440 uL (range: 60-4000 uL), using the QIAamp Circulating Nucleic Acids kit (Qiagen, Venlo, the Netherlands). Subsequently, the presence of *ESR1* mutations was investigated using the Ion Torrent™ Oncomine™ cfDNA Assay for breast cancer, according to protocols and consumables provided by the manufacturer (Life Technologies, Thermo Fisher Scientific, Carlsbad, California, US). The Oncomine™ cfDNA Assay for breast cancer is a targeted panel covering multiple hotspots in 10 genes important in breast cancer (*TP53, PIK3CA, ESR1, ERBB2, ERBB3, AKT1, SF3B1, KRAS, EGFR*, and *FBXW7*). For *ESR1* the following hotspot mutations are included within this panel: p.D538G, p.Y537S/C/N, p.E380Q, p.V392I, and p.S463P. This NGS panel is equipped with unique molecular identifiers (UMIs) to enable sensitive detection of truly mutated copies. By providing simultaneous information on multiple mutations, the use of a NGS panel enabled us to investigate *ESR1* polyclonality and also *ESR1* dynamics in plasma. Library preparation for this panel requires 13 uL of DNA input. We used a maximum of 50-ng DNA and if the total DNA yield did not exceed 10 ng, the sample was vacuum dried to a total amount of 13 uL, which was then used for library preparation. Molecular coverage was defined as the count of unique molecules and known hotspot variants were analyzed if they were detected in at least three unique molecules. For follow-up samples, also mutations detected in < 3 molecules were assigned as true variant if the mutation was already identified in a baseline sample. To ensure a sufficient coverage, a total of at least 500 unique molecules had to be sequenced. If this was not reached during the first sequencing run, new libraries were prepared and sequenced and data of the two sequencing runs were summed. This resulted in a theoretical lower limit of detection (LOD) of a variant allele frequency (VAF) of 0.006% (3/500). *TP53* mutations were not included in the analysis as we could not exclude the possibility that they had resulted from clonal hematopoiesis [14] and are, therefore, not tumor specific. Germline DNA was not collected within this study. For analysis of ctDNA dynamics, mutations were only included if the molecular coverage at C2 was sufficient to detect the mutation at the baseline frequency.

### Assessment of endpoints

The primary endpoint of the ATX study was investigator-assessed PFS, defined as the time from randomization to disease progression or death from any cause, whichever came first. Tumor assessment was performed according to Response Evaluation Criteria in Solid Tumors (RECIST 1.0) every 3 months by computed tomography or magnetic resonance imaging. Secondary endpoints were overall survival (OS), defined as time from randomization to death from any cause, and objective response rate (ORR). ORR was defined as the percentage of complete and partial response confirmed after a minimum of four weeks after first being reported.

### Statistical analysis

The primary objective of this study was to investigate whether patients with an *ESR1* mutation benefit from taxane-based chemotherapy. Therefore, we hypothesized that taxane-based treatment would increase the PFS at 6 months when compared to historical trials with fulvestrant, the current standard of treatment in patients with an *ESR1* mutation. The PFS percentage at 6 months after start of treatment with fulvestrant in this specific patient category is approximately 40% [6]. For this study, we proposed that a PFS at 6 months of approximately 75% would justify further testing of chemotherapy versus fulvestrant in a phase III randomized trial [12]. To test this hypothesis, we used a Simon two-stage design with p0 = 0.4 and p1 = 0.75 with a power of 0.8 and a type 1 error rate of 0.05. As such, at least 8 out of 14 patients with an *ESR1* mutation had to be free of progression at 6 months to conclude that chemotherapy is sufficiently promising in this patient group to warrant further investigation. Using an exploratory analysis, we compared PFS and OS in patients with and without a detectable *ESR1* mutation. Descriptive statistics were calculated for variables of interest. Wilcoxon signed-rank test was performed for univariate analyses of continuous variables and a Fisher exact test or chi-square test was used for categorical variables. The duration of time to event was estimated using the Kaplan–Meier method. Statistical tests were two-sided and considered statistically significant when *p* < 0.05. IBM SPSS STATISTICS 25 (ICM Corp, Armonk, NY) was used for survival analysis and descriptive statistics. Prism™ software (GraphPad Software, La Jola, Ca) was used for the statistical analyses of ctDNA dynamics.

## Results

### ESR1 mutation analysis

**Fig. 1:**
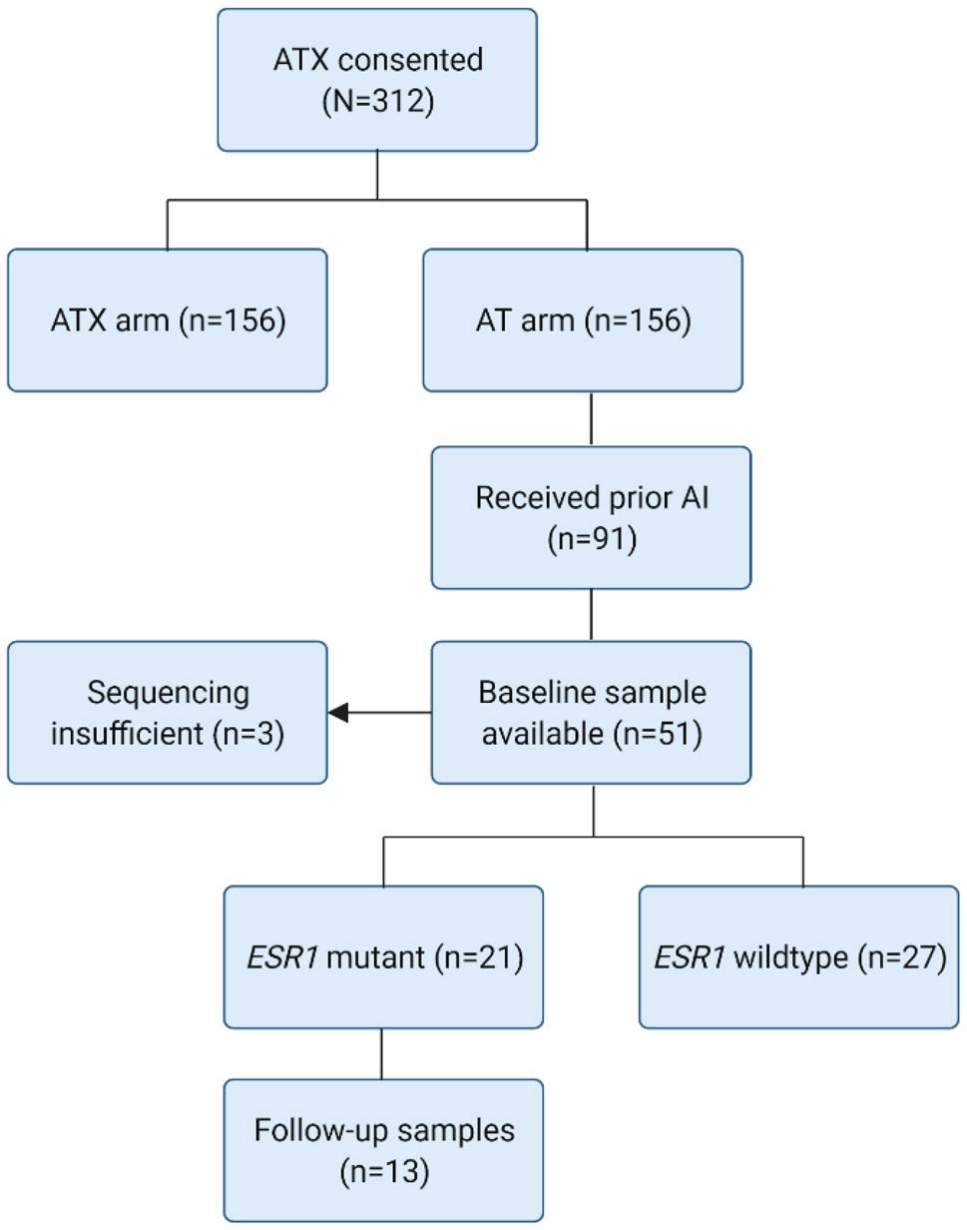
CONSORT diagram demonstrating samples analyzed in the ATX trial

**Fig. 2:**
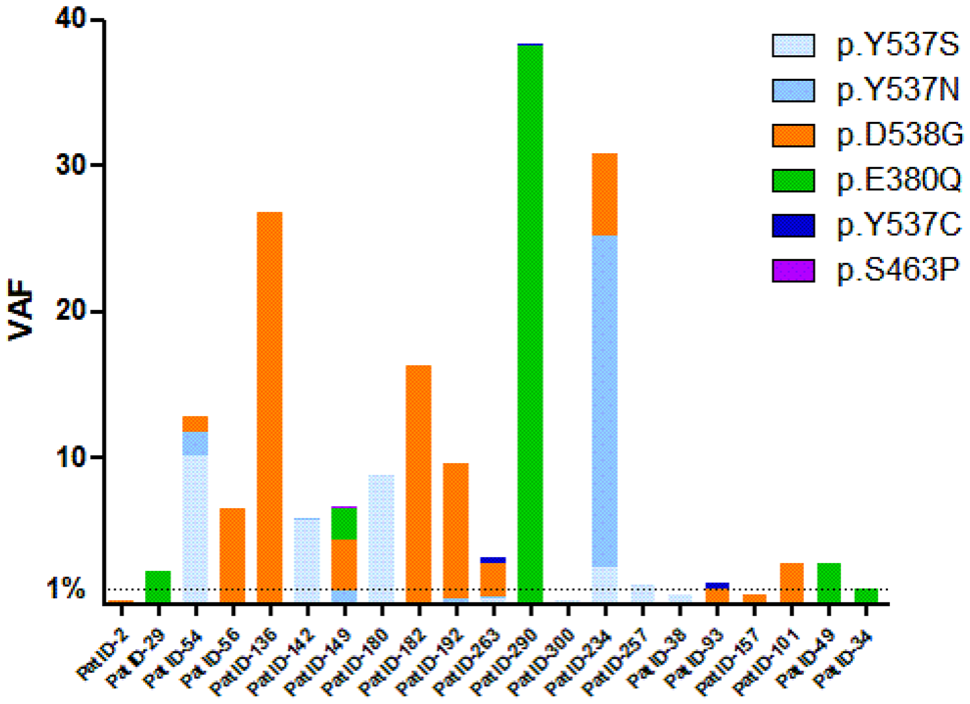
*ESR1* variants in patients with detectable *ESR1* mutations

In the ATX trial, 156 patients were randomized to paclitaxel/bevacizumab (AT arm), of whom 85% were ER-positive. Of those, 91 patients received prior treatment with an AI in the adjuvant and/or metastatic setting. Residual baseline plasma samples were available of 51 patients (56%), since plasma had already been used for other purposes [9, 10]. Samples were subjected to NGS. Three samples did not reach a total molecular coverage of ≥ 500 molecules, despite duplicate library preparation and sequencing and were, therefore, excluded from the analysis (Fig. 1). In total 48 patients were included, of which 21 (44%) had a detectable *ESR1* mutation. Adjuvant endocrine treatment was not different among patients with *ESR1* mutant and *wildtype* tumors (*p* = 0.28), whereas patients with an *ESR1* mutation tended to have received palliative endocrine treatment more often 86% vs 67% (*p* = 0.19). The median cfDNA concentration was 42-ng/mL plasma (range: 10–125 ng/mL) among patients with *ESR1* mutant tumors and 24-ng/mL plasma (range: 6–133 ng/mL) among patients with *ESR1 wildtype* tumors (*p* = 0.1). Of the patients with a detectable *ESR1* mutation, eight patients (38%) had multiple *ESR1* mutations (Fig. 2).

### ESR1 mutations and PFS

**Fig. 3:**
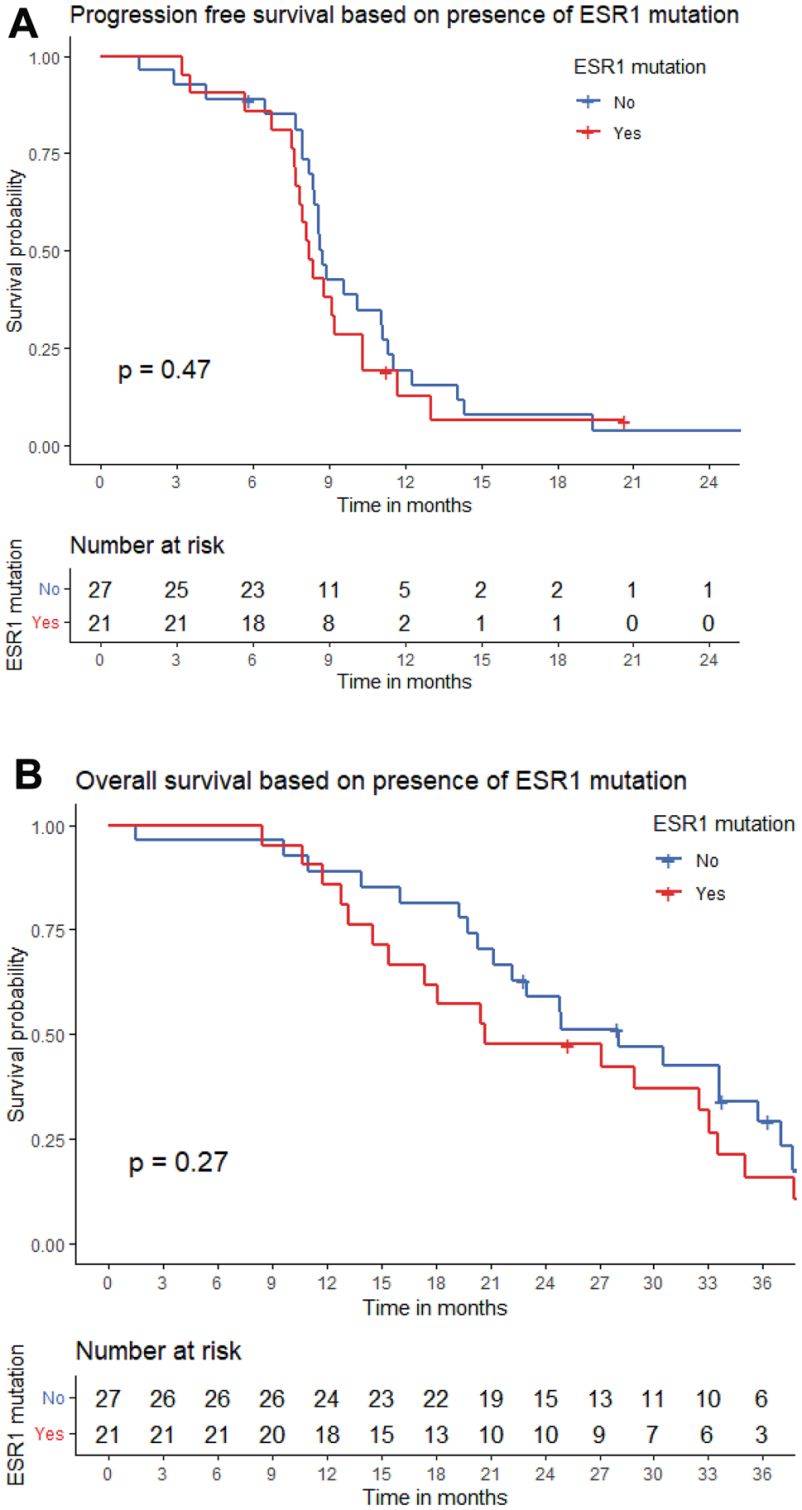
**A** Progression-free survival in patients treated with paclitaxel/bevacizumab by *ESR1* mutation status. **B** Overall survival in these patients by *ESR1* mutation status

One patient died before progression and three patients started other systemic therapy before radiological progression. PFS at 6 months was 86% (18/21) in patients with an *ESR1* mutation detected and 85% (23/27) in wildtype *ESR1* patients. In patients with a detectable *ESR1* mutation, median PFS was 8.2 months (95% CI 7.6–8.8 months) and in wildtype *ESR1* patients, median PFS was 8.7 months (95% CI 8.3–9.2 months, log rank *p* = 0.47, Fig. 3A). When split by the median *ESR1* VAF, PFS was not different between patients with a *ESR1* VAF above the median (median 8.1 months, 95% CI 7.8–8.5 months) and a *ESR1* VAF below the median (median 8.4 months, 95% CI 7.2–9.6 months, log rank *p* = 0.581). Also, PFS was not significantly different among patients with multiple *ESR1* mutations (median: 7.5 months, 95% CI 5.2–9.8 months) versus patients without or one *ESR1* mutation(s) (median 8.6 months, 95% CI 8.2–9.1 months, log rank p = 0.35). We did not analyze a possible relation of the presence of specific *ESR1* variants with outcome as the numbers were too low Table 1.

**Table 1:** Baseline characteristics associated with *ESR1* mutation detection in baseline ctDNA

Characteristic		*ESR1 wildtype n* = 27	*ESR1* mutant *n* = 21	
Age	Median (range)	57 (43–65)	58 (39–68)	*p* = 0.26
ECOG PS				
0	0	16 (59%)	8 (38%)	
1	1	11 (41%)	13 (62%)	*p* = 0.24
Disease-free interval <12 months	n (%)	6 (22%)	3 (14%)	*p* = 0.71
Prior adjuvant hormonal therapy				
None	n (%)	13 (48%)	12 (57%)	
Tamoxifen	n (%)	3 (11%)	4 (19%)	
AI	n (%)	4 (15%)	0 (0%)	
Tamoxifen+AI	n (%)	7 (26%)	5 (24%)	*p* = 0.28
Prior palliative hormonal therapy	n (%)	18 (67%)	18 (86%)	*p* = 0.19
Prior neoadjuvant chemotherapy	n (%)	14 (52%)	11 (52%)	*p* = 1
cfDNA concentration ng/mL	Median (range)	24 (6–133)	42 (10–125)	*p* = 0.1

### ESR1 mutations, OS, and objective response rate

We subsequently investigated the association between *ESR1* mutation status and OS, with a total of 42 deaths. In patients with a detectable *ESR1* mutation, median OS was 20.7 months (95% CI 6.6–33.7 months), whereas in wildtype *ESR1* patients, median OS was 28.1 months (95% CI 19.3–36.9 months, log rank *p* = 0.27, Fig. 3B). When split by the median *ESR1* VAF, OS was not different between patients with a high *ESR1* VAF (median 20.7 months, 95% CI 9.1–32.3 months) and a low *ESR1* VAF (median 17.4 months, 95% CI 6.9–30.9 months, log rank *p* = 0.7). Interestingly, OS was significantly shorter in patients with multiple *ESR1* mutations (median: 14.6 months, 95% CI 8.1–21.0 months) when compared to those without or with one *ESR1* mutation (median 28.9 months, 95% CI 20.4–37.5 months, *p* = 0.003, Figure S1). Moreover, the total number of mutations in any of the ten genes analyzed was significantly correlated with overall survival in univariate cox proportional hazard regression analysis (HR 1.26, 95% CI 1.04–1.54, *p* = 0.017). In the *ESR1 wildtype* group, one patient (4%) had a complete response to treatment, whereas none of the patients in the *ESR1* mutant group had a complete response. Overall, the ORR was lower in patients with an *ESR1* mutation (40%) than in wildtype *ESR1* patients (65%), but this was not significant (*p* = 0.136, Table 2).

**Table 2:** Objective response according to RECIST by *ESR1* mutation status

	*ESR1 wildtype* *n* = 27	*ESR1* mutant *n* = 21
CR	1 (4%)	0 (0%)
PR	16 (59%)	8 (38%)
SD	8 (30%)	11 (52%)
PD	1 (4%)	1 (5%)
NE	1 (4%)	1 (5%)

*CR* complete response, *PR* partial response, *SD* stable disease, *PD* progressive disease, *NE*, not evaluated

### ctDNA dynamics

**Fig. 4:**
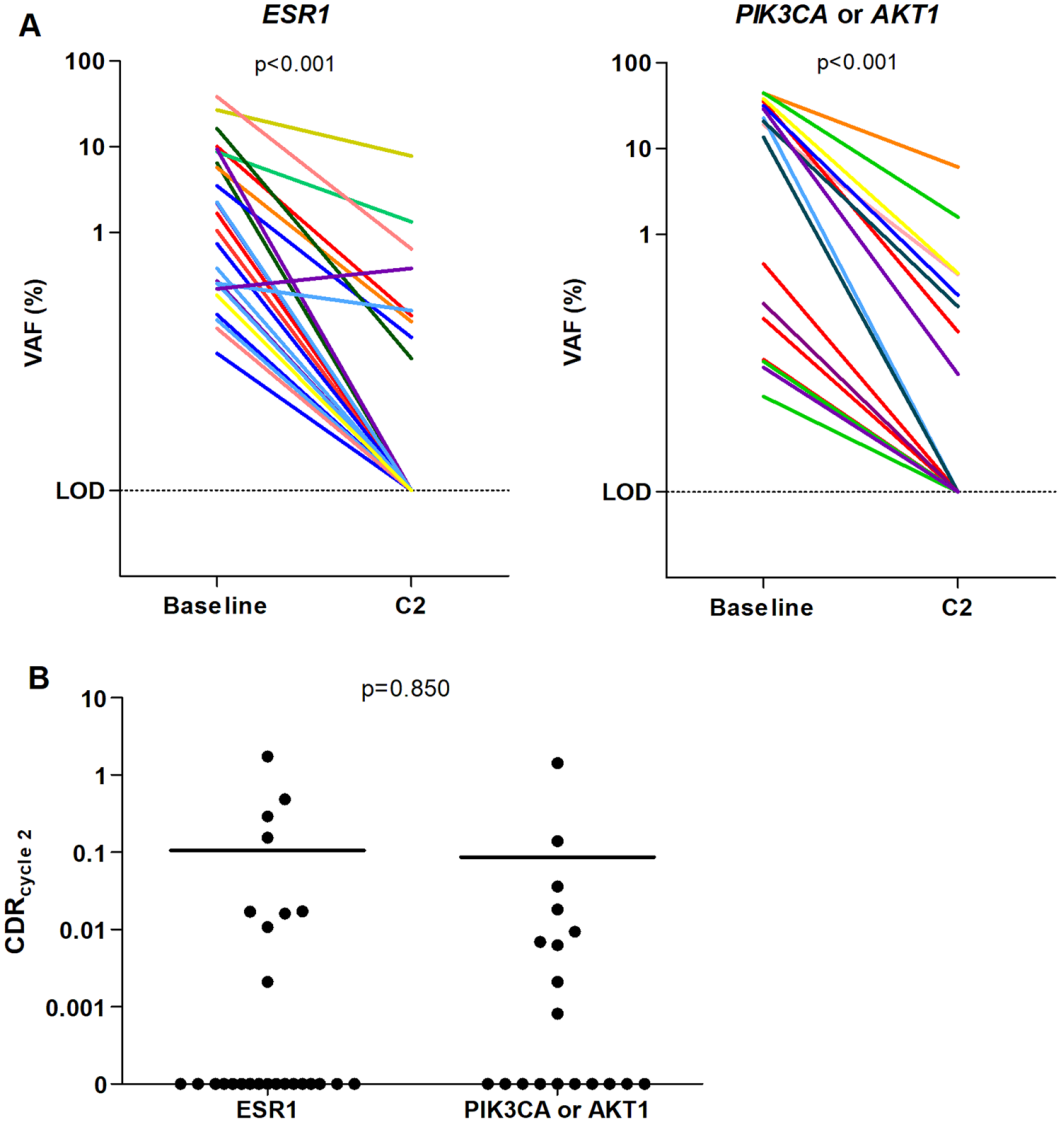
**A** Dynamics of the VAF of *ESR1* and *PIK3CA* or *AKT1* mutations between baseline and cycle 2, day 1, and differences between the VAF were calculated using the Wilcoxon signed-rank test. Connecting lines are colored per patient. **B** CDR of the VAF from *ESR1* mutation vs *PIK3CA* or *AKT1* mutations. Differences between the CDR were calculated using the Wilcoxon signed-rank test. Line at median

Of the 21 patients with an *ESR1* mutation, follow-up samples were available from 13 patients (62%). Of those, 11 patients also had a *PIK3CA* or *AKT1* mutation. At C2, *ESR1* mutations were undetectable in four patients (31%). In total, 24 *ESR1* and 18 *PIK3CA* or *AKT1* mutations were identified in baseline samples and assessed in follow-up samples. Those *PIK3CA* and *AKT1* mutations were considered as a surrogate marker for ctDNA load and used to calculate whether *ESR1* mutant subclones showed a differential response to treatment. Of the 24 individual *ESR1* mutations, 16 (67%) were not detected in follow-up samples. Additionally, of the 20 *PIK3CA* or *AKT1* mutations, 9 (45%) were not detected in follow-up samples (Fig. 2A). We defined the circulating DNA ratio (CDR) as the ratio of mutation abundance on treatment (C2) relative to baseline, in which CDR represents the ratio of ctDNA levels at C2 to ctDNA levels at baseline. All patients had a CDR < 1 indicating a fall in ctDNA. The CDR was not different between *ESR1* mutations and *PIK3CA* or *AKT1* mutations (*p* = 0.547, Fig. 2B), suggesting there was no differential response to treatment in *ESR1* mutant subclones versus *PIK3CA* or *AKT1* mutant subclones. The results were similar for mutant copies/mL plasma (Figure S2). Because of the low patient number from whom follow-up samples were available, analysis of CDR and outcome to paclitaxel/bevacizumab was not performed as shown in Fig. 4.

## Discussion

To investigate the clinical impact of *ESR1* mutations in cfDNA on the efficacy of chemotherapy in metastatic ER+/HER2- breast cancer patients, we analyzed plasma samples from advanced ER+ breast cancer patients that were treated with first-line paclitaxel/bevacizumab in the ATX trial. We demonstrated that paclitaxel/bevacizumab might be an effective treatment strategy in patients independent of *ESR1* mutations status. Based on comparison with literature, which suggests the PFS at 6 months after treatment with fulvestrant is 40% [6], treatment with paclitaxel/bevacizumab has a superior PFS at six months of 85% regardless *ESR1* mutation status. The results of this retrospective, exploratory study suggest that the efficacy of chemotherapy is retained in patients with one or more *ESR1* mutations, although our study was not powered to detect a difference in survival between patients with *ESR1* mutations and wildtype *ESR1* patients.

We observed that chemotherapy resulted in a decline of *ESR1* mutant ctDNA after one cycle of chemotherapy. For the two patients with confirmed progression at 6 months after treatment initiation, we did not have a C2 sample available to investigate whether ctDNA dynamics could have predicted this outcome. In the majority of *ESR1* mutant patients, the mutation could not be detected after one cycle of treatment. Although we used plasma samples containing an unknown amount of *wildtype* contamination, we observed a fall in ctDNA levels of both *ESR1* and *PIK3CA* or *AKT1* mutations after 1 cycle of treatment, suggesting that this fall in ctDNA load represents a decreased tumor load rather than pre-analytical variation. Additionally, we found that there was no difference in suppression of *ESR1* mutations, which were often subclonal, when compared to *PIK3CA* or *AKT1* mutations, suggesting the anti-proliferative effect of chemotherapy does not differentially affect cells that have acquired an *ESR1* mutation.

We found that the presence of multiple *ESR1* mutations, but not single *ESR1* mutations, was associated with an impaired OS, although in a small set of patients. *ESR1* mutations were previously identified as prognostic biomarker in the BOLERO-2 study [5], as well as in the EFECT and SoFEA trial [21]. The prognostic value of having polyclonal *ESR1* mutations was also observed in the BOLERO-2 trial [5]. Patients with polyclonal *ESR1* mutations in ctDNA might have tumors that are heterogeneous and, therefore, more likely to consist of other resistant subclones besides *ESR1*. To substantiate this finding, we took the total number of mutations in any of the ten genes that were analyzed as a surrogate for heterogeneity and demonstrated that this total number of mutations was significantly correlated with overall survival. This finding highlights the importance of understanding tumor heterogeneity in a non-invasive manner and emphasize approaches to target or even to prevent this heterogeneity.

Previous studies have suggested that *ESR1* variants differentially impact sensitivity to treatment. In analyses from the PALOMA-3 and SoFEA study, patients harboring the *ESR1* p.Y537S had the shortest PFS on fulvestrant and this variant was enriched after treatment with fulvestrant with or without CDK4/6-i (16). Also in patients treated with everolimus and exemestane, *ESR1* p.Y537S tended to be negatively associated with PFS and OS [7]. As our study was limited by the sample size, we did not perform subgroup analysis for specific *ESR1* variants. In general, the *ESR1* variant with the highest VAF was the mutation that remained detectable in patients with polyclonal *ESR1* mutations, suggesting the decline in VAF is a reflection of decreasing tumor load rather than a differential response.

As a hypothesis, detection of *ESR1* mutations might be useful to determine whether sensitivity to endocrine treatment might be restored after anti-proliferative agents such as CDK4/6-i or chemotherapy. The CHRONOS study has demonstrated that patients with metastatic colorectal cancer [17], who have progressed due to *RAS/RAF* mutations after previous response to anti-EGFR treatment and who lacked detectable *BRAF* or *RAS* mutations in ctDNA after progression to the last anti-EGFR-free regimen, could be successfully re-challenged with anti-EGFR treatment. As such, it might be investigated whether endocrine-resistant advanced breast cancer patients in whom *ESR1* mutations become undetectable after chemotherapy can be successfully re-introduced to endocrine treatment.

This retrospective study contains several flaws. The analysis of the impact of the *ESR1* mutational status on outcome of chemotherapy was limited due to the relatively low sample size at baseline and the number of patients with samples available at C2. The small sample size also hampered us from conducting various subanalyses investigating the differential impact of *ESR1* variants or variants in other genes on outcome. Also, we were not able to investigate the effect of ctDNA dynamics on outcome. As such, our findings are exploratory and should be interpreted with caution. The lack of plasma samples at progression precluded the question, whether chemotherapy can lead to definite clearance of *ESR1* mutations enabling re-challenge with endocrine treatment. Furthermore, bevacizumab is no longer added to taxane-based chemotherapy in current practice. The PFS benefit of adding bevacizumab to taxane-based treatment [12] could not be validated in later studies and did not translate into OS benefit [13, 15] resulting in the FDAs withdrawal of the breast cancer indication for bevacizumab. Although we could not propose a mechanism by which VEGF inhibition could have affected the cytostatic effect of paclitaxel on *ESR1* mutant cells, the effect of this limitation on the results of this study remain unknown. The limited sample size of this study might also be an explanation for our finding that there was no statistical difference in receipt of prior endocrine treatment between *ESR1* mutant and wildtype patients, while literature indicates that *ESR1* mutations mainly occur after treatment with an AI in the metastatic setting [1, 16, 18, 20]. In conclusion, we found no association between the presence of *ESR1* mutations in baseline ctDNA and PFS in patients treated with paclitaxel/bevacizumab. Therefore, the results of this retrospective, exploratory study suggest that the efficacy of chemotherapy is retained in patients with one or more *ESR1* mutations. We further found that having polyclonal disease was associated with impaired OS. Lastly, we found that in some patients, *ESR1* mutations become undetectable after one cycle of chemotherapy. Whether this results in restoration of endocrine sensitivity remains to be investigated.

## Supplementary Information

Below is the link to the electronic supplementary material.

Supplementary file1 (DOCX 1033 KB)

## Data Availability

The datasets used and/or analyzed during the current study are available from the corresponding author on reasonable request.
