# Supplementary material for: Plasma ESR1 mutations and outcome to first-line paclitaxel and bevacizumab in patients with advanced ER-positive/HER2-negative breast cancer
Source: Breast Cancer Res Treat. 2023 May 25;200(2):271–9. doi: 10.1007/s10549-023-06965-5 (PMC10241671; doi:10.1007/s10549-023-06965-5)
Supplement: Supplementary file 1 — Supplementary file1 (DOCX 1033 KB) [file 10549_2023_6965_MOESM1_ESM.docx]

**Supplemental figures**

**Figure S1.** Overall survival in patients treated with paclitaxel/bevacizumab based on the presence of multiple *ESR1* mutations


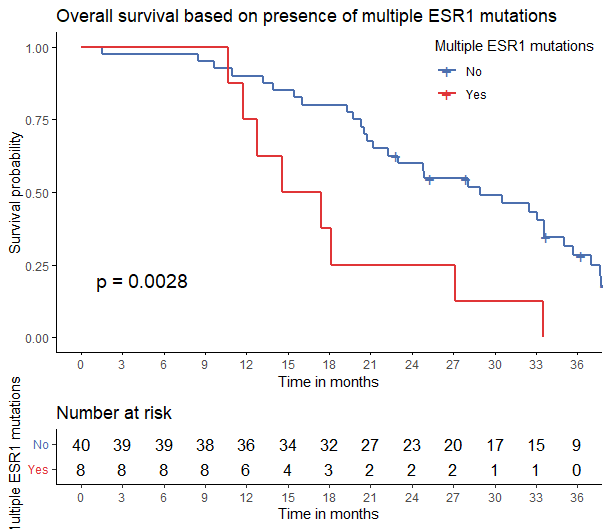


**Figure S2. A.** Dynamics of the mutant *ESR1* copies and mutant *PIK3CA* or AKT1 copies between baseline and cycle 2, day 1, p value Wilcoxon signed-rank test. **B.** CDR of the mutant copies from *ESR1* mutation vs *PIK3CA* or *AKT1* mutations. P value Wilcoxon signed-rank test. Line at median. Connecting lines are colored per patient.

**
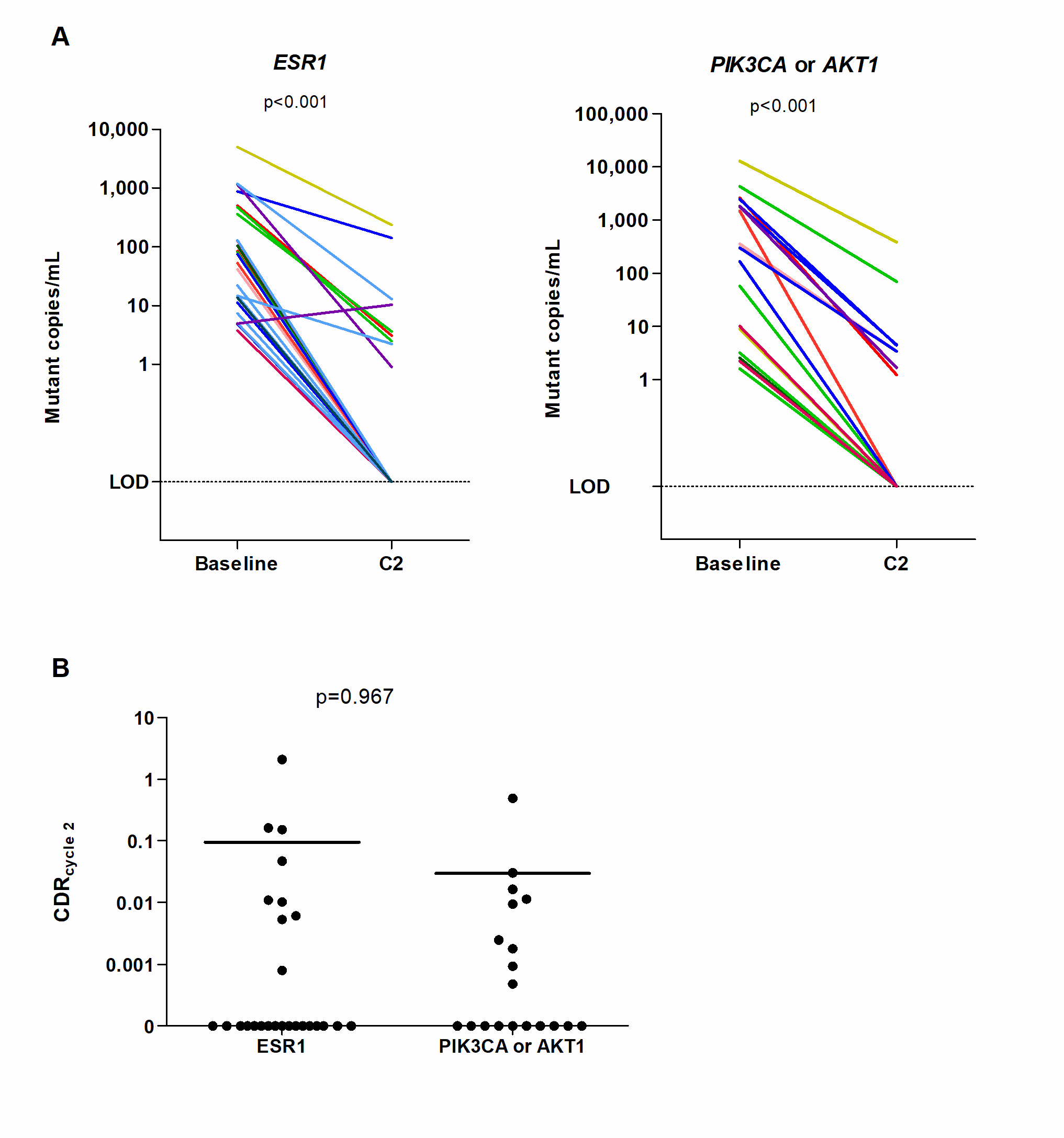
**
